# Perinatal Opioid Exposure Primes the Peripheral Immune System Toward Hyperreactivity

**DOI:** 10.3389/fped.2020.00272

**Published:** 2020-06-26

**Authors:** Jessie Newville, Jessie R. Maxwell, Yuma Kitase, Shenandoah Robinson, Lauren L. Jantzie

**Affiliations:** ^1^Department of Neurosciences, University of New Mexico School of Medicine, Albuquerque, NM, United States; ^2^Departments of Pediatrics, University of New Mexico School of Medicine, Albuquerque, NM, United States; ^3^Division of Neonatal-Perinatal Medicine, Department of Pediatrics, Johns Hopkins University School of Medicine, Baltimore, MD, United States; ^4^Division of Pediatric Neurosurgery, Department of Neurosurgery, Johns Hopkins University School of Medicine, Baltimore, MD, United States; ^5^Department of Neurology, Johns Hopkins University School of Medicine, Baltimore, MD, United States; ^6^Department of Neurology, Kennedy Krieger Institute, Baltimore, MD, United States

**Keywords:** methadone, lymphocyte, PBMC, SPIHR, blood mononuclear cell, neuroinflammation, neonatal abstinence syndrome, prenatal

## Abstract

The increased incidence of opioid use during pregnancy warrants investigation to reveal the impact of opioid exposure on the developing fetus. Exposure during critical periods of development could have enduring consequences for affected individuals. Particularly, evidence is mounting that developmental injury can result in immune priming, whereby subsequent immune activation elicits an exaggerated immune response. This maladaptive hypersensitivity to immune challenge perpetuates dysregulated inflammatory signaling and poor health outcomes. Utilizing an established preclinical rat model of perinatal methadone exposure, we sought to investigate the consequences of developmental opioid exposure on *in vitro* activation of peripheral blood mononuclear cells (PBMCs). We hypothesize that PBMCs from methadone-exposed rats would exhibit abnormal chemokine and cytokine expression at baseline, with exaggerated chemokine and cytokine production following immune stimulation compared to saline-exposed controls. On postnatal day (P) 7, pup PMBCs were isolated and cultured, pooling three pups per *n*. Following 3 and 24 h, the supernatant from cultured PMBCs was collected and assessed for inflammatory cytokine and chemokine expression at baseline or lipopolysaccharide (LPS) stimulation using multiplex electrochemiluminescence. Following 3 and 24 h, baseline production of proinflammatory chemokine and cytokine levels were significantly increased in methadone PBMCs (*p* < 0.0001). Stimulation with LPS for 3 h resulted in increased tumor necrosis factor (TNF-α) and C-X-C motif chemokine ligand 1 (CXCL1) expression by 3.5-fold in PBMCs from methadone-exposed PBMCs compared to PBMCs from saline-exposed controls (*p* < 0.0001). Peripheral blood mononuclear cell hyperreactivity was still apparent at 24 h of LPS stimulation, evidenced by significantly increased TNF-α, CXCL1, interleukin 6 (IL-6), and IL-10 production by methadone PMBCs compared to saline control PBMCs (*p* < 0.0001). Together, we provide evidence of increased production of proinflammatory molecules from methadone PBMCs at baseline, in addition to sustained hyperreactivity relative to saline-exposed controls. Exaggerated peripheral immune responses exacerbate inflammatory signaling, with subsequent consequences on many organ systems throughout the body, such as the developing nervous system. Enhanced understanding of these inflammatory mechanisms will allow for appropriate therapeutic development for infants who were exposed to opioids during development. Furthermore, these data highlight the utility of this *in vitro* PBMC assay technique for future biomarker development to guide specific treatment for patients exposed to opioids during gestation.

## Introduction

The incidence of opioid abuse in the United States has steadily increased since 2000, today reaching epidemic proportions (1, 2). The crisis is illustrated by data demonstrating that the number of opioid-related hospitalizations increased by 64%, and the number of deaths due to opioid overdose increased by 27% between the years of 2005 and 2014 (3, 4). Mirroring the national trend, opioid use by pregnant women has escalated to alarming rates (5). Opioid use in this population increased 5-fold from 2000 to 2009 (6), and the prevalence of women with opioid use disorders at delivery hospitalizations quadrupled between 1999 and 2014 (7).

Prenatal opioid exposure results from maternal use or abuse of illicit opioids, such as heroin, and prescription opioids including oxycodone, hydrocodone, morphine, codeine, and fentanyl (8). In addition, the maternal use of buprenorphine or methadone, two synthetic opioids commonly used in opioid maintenance therapy for individuals suffering from opioid use disorder (9, 10), also contribute to prenatal opioid exposure. The growing rate of women using opioids during pregnancy has led to an increase in adverse neonatal outcomes (11, 12). Recent studies showing an association between opioid use during pregnancy and poor health outcomes for both pregnant women and infants highlight prenatal opioid exposure as a serious public health concern (13, 14). Opioid-exposed infants represent an extremely vulnerable patient population (15), with 50–80% experiencing neonatal abstinence syndrome (16). Indeed, prenatal opioid exposure is associated with increased risk of fetal growth restriction, preterm birth, and lifelong motor and cognitive deficits (17–25). The devastating consequences of opioid exposure on the physical health and developmental outcomes of exposed children strengthen the need to advance scientific understanding of the underpinnings of opioid-induced neural injury and to advance biomarker development in this patient population.

Insult during the prenatal period affects ongoing developmental processes in the fetus, leading to lifelong consequences and health challenges. Both the central nervous system (CNS) and the immune system undergo complex and incremental steps toward maturation during gestation (26–28). New advances in molecular neuroscience have begun to elucidate the importance of the multifaceted interplay of central and peripheral immune system in regulating and supporting ongoing brain development. Moreover, these advances highlight the neurodevelopmental consequences of perinatal immune activation following perinatal insult (29–33). The findings from both clinical and preclinical studies implicate perinatal immune activation in the pathophysiology of numerous neurodevelopmental disorders, such as cerebral palsy, autism spectrum disorders, Down syndrome, and fetal alcohol spectrum disorders (33–41).

Previously, we reported evidence of neural injury and reduced cognitive functioning in a model of prenatal opioid exposure, with multiple assays reflecting significant neuroinflammation (42). In the aforementioned study, analysis of serum inflammatory cytokine expression of opioid-exposed animals compared to saline-exposed controls demonstrated elevated levels of interleukin 1β (IL-1β), tumor necrosis factor α (TNF-α), IL-6, and C-X-C motif chemokine ligand 1 (CXCL1), indicating systemic inflammatory response syndrome induced by opioid exposure. Additionally, initial *in vitro* assessment of isolated PBMC from opioid-exposed animals challenged with lipopolysaccharide (LPS) suggested heightened immune reactivity and immune priming toward exaggerated responses to stimuli (42).

Here, we extend our investigation of opioid-induced inflammation by thoroughly defining the peripheral immune signaling and reactivity of opioid-exposed PBMCs using an established *in vitro* assay and biomarker platform (35, 37, 43–50). These data enhance the understanding of important inflammatory mechanisms, an essential step to inform future development of appropriate therapeutic interventions for infants who are exposed to opioids during gestation.

## Materials and Methods

### Animals

Sprague–Dawley rat dams and litters were maintained in a 12-h dark–light cycle (lights on at 0800 h), temperature, and humidity-controlled facility with food and water available *ad libitum*. All experiments were performed in strict accordance with protocols approved by the Institutional Animal Care and Use Committee at the University of New Mexico Health Sciences Center. Protocols were developed and performed consistent with National Research Council and ARRIVE guidelines (51).

### Opioid Administration

**Figure 1:**
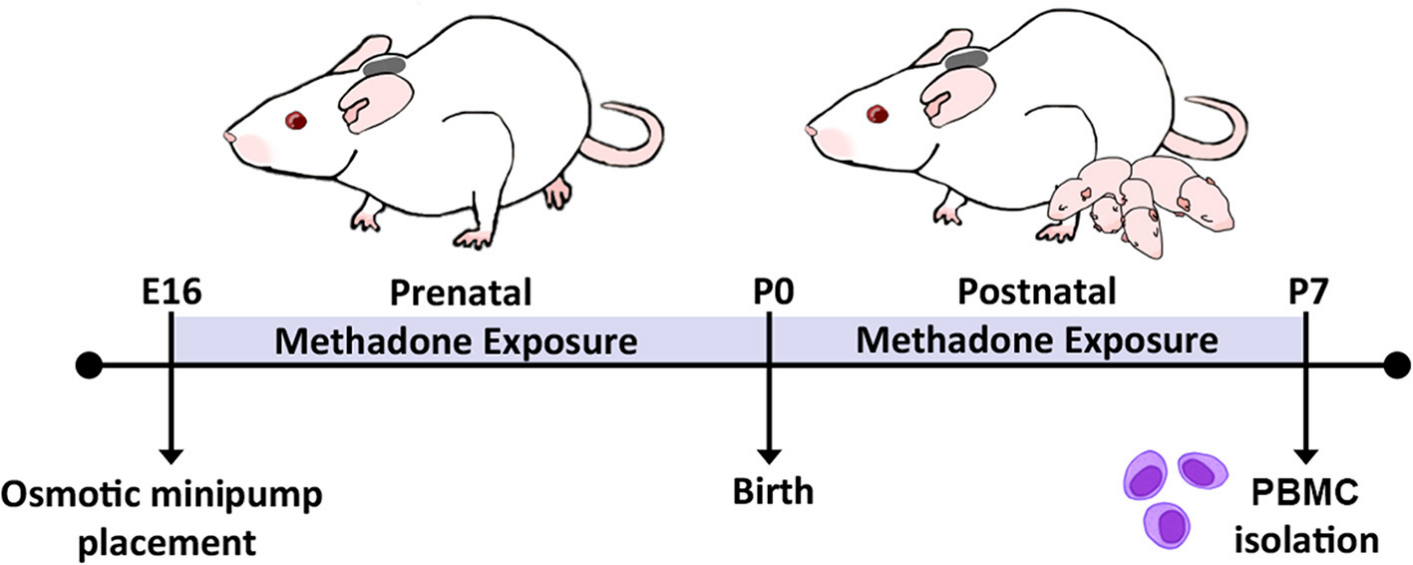
Experimental timeline. Perinatal methadone exposure was accomplished by minipump implantation on E16, permitting pup exposure to methadone during critical stages of immune and neurological maturation. On P7, PBMCs from methadone- or saline-exposed pups were isolated for culture and biochemical analysis.

Methadone is a full μ-, δ-, and κ-opioid receptor agonist, similar to heroin, morphine, and fentanyl, whereas buprenorphine is a partial μ-opioid receptor agonist and κ-opioid receptor antagonist (52). The use of methadone in our experiments allows us avoid differential pharmacology related to partial antagonism and study the effects from of full agonism at the predominant opioid receptor subtypes. As previously published (42), osmotic minipumps (model 2004; Alzet, Cupertino, CA, USA) primed with 12 mg/kg of methadone or normal saline were implanted in embryonic day (E) 16 timed-pregnant rat dams (Charles River Laboratories, Wilmington, MA, USA) (Figure 1). Implantation on E16 allows *in utero* opioid exposure from E16 to birth and postnatal opioid exposure via milk from birth to postnatal day (P) 7 (blood collection). These minipumps allow for continual infusion of methadone or saline at a rate of 0.25 μL per hour for a maximum of 28 days. Under isoflurane-induced anesthesia, dams underwent a minipump placement procedure. Subcutaneous minipump placement was achieved by transverse 1.5-cm incision. The subcutaneous area was opened by careful blunt dissection, and the prefilled, primed osmotic minipump was placed in the opened space. Following closure of the incision with sutures, dams were then returned to their respective home cages, where their recovery was closely monitored. When pups were born on E22, they then received methadone through milk ingestion. Postnatal methadone exposure was confirmed by measuring the concentration of methadone in dam and offspring urine (42). As previously reported, this paradigm of opioid exposure results in significant pup weight loss at the neonatal and perinatal period. Opioid exposure via 12 mg/kg minipump results in a significant 10% reduction in offspring weight at P1 and 23% reduction in weight by P21 compared to saline exposed controls (42). These preclinical data reflect data from clinical studies showing that infants of mothers who exclusively used opioids suffered from a 2 to 10% decrease in birth weight compared to healthy controls (53, 54). Further, another study found that infants of mothers on methadone replacement therapy suffered a 19% reduction in birth weight compared to age-matched controls (55). Thus, this model replicates the systemic consequences of extended prenatal opioid exposure observed in human infants.

### Peripheral Blood Mononuclear Cell Isolation

At P7, rats are developmentally equivalent to human infants between approximately 32 and 38 weeks' gestation (56–62). From P7 to P10 in rats and 36–40 weeks' gestation in humans (term infant), overall brain growth peaks while important neural developmental processes, such as gliogenesis and expansion of axonal and dendritic density, occur (60, 63–65, 65–69). During this same developmental period, consolidation of the immune system in humans and rats occurs, whereby the functional capacity of immune cells evolves, and the number of circulating leukocytes, neutrophils, and monocytes increases (26–28, 60, 70–72). At P7, PBMC isolation was performed as previously published (41). Venous blood was collected from the right atrium of deeply anesthetized P7 pups and pooled across three animals in pyrogen-free K2 EDTA Vacutainer tubes (366643; Becton Dickson, Franklin Lakes, NJ, USA). Each n represents PBMCs isolated from blood pooled across three separate animals. In this study, equal numbers of male and female pups were used throughout. Peripheral blood mononuclear cells were isolated by Ficoll gradient separation (37), whereby equal volumes of peripheral blood and RPMI 1640 media (Gibco, Waltham, MA, USA) were combined and layered atop 3 mL of Ficoll-Plaque Plus (17144002; GE Healthcare, Chicago, IL, USA) within sterile 15-mL conical tubes. Following centrifugation at 400 *g* for 30 min at room temperature, the PBMC cell layer was transferred to a new centrifuge tube and resuspended in three volumes of RPMI media. Two wash cycles were performed, consisting of centrifugation at 400 *g* for 10 min at room temperature, disposal of the supernatant, and resuspension of the pellet in three volumes of RPMI media. Isolated PBMCs resuspended in media were plated in triplicates at a density of 1 × 10^6^ cells/mL on 3.5-cm Petri dishes.

### Peripheral Blood Mononuclear Cell Treatment With LPS

Plated PBMCs from saline and methadone groups were stimulated with 10, 50, or 100 ng/mL of LPS to generate a dose response curve. Based on TNF-α secretion levels at 3 h, we determined that stimulation with 100 ng of LPS was ideal to produce a robust PBMC secretory response in both treatment groups. Therefore, consistent with previous studies, 100 ng of LPS was used for LPS challenge experiments (41, 45, 73). Supernatant samples were collected at 3 and 24 h in sterile 2-mL Eppendorf tubes, snap frozen on dry ice, and stored at −80°C until biochemical analysis.

### Multiplex Electrochemiluminescent Immunoassay

To capture the secretory activity of PBMCs prior to protein synthesis, and then after protein synthesis, the supernatants from plated PBMCs were assayed at 3 and 24 h, respectively. Subsequently, secreted cytokine and chemokine expression was quantified using a V-PLEX Proinflammatory Panel 2 Rat Kit (K15059D; Meso Scale Diagnostics, Rockville, MD, USA) created to detect levels of interferon γ, IL-1β, IL-4, IL-5, IL-10, IL-13, IL-6, CXCL1, and TNF-α. The V-PLEX multielectrochemiluminescent immunoassay (MECI) was performed according to manufacturer instructions with <5% interassay variation. Specifically, supernatants from cultured PBMCs were diluted 1:4 and, together with prepared standards, were loaded in duplicate onto the manufacturer-provided blocked and washed 96-well plates. Then, following a series of washes and incubation with the antibody detection solution, plates were washed and loaded with the manufacturer-provided Read Buffer and read on a Quickplex SQ 120 Imager. Here, we report data on the levels of TNF-α and CXCL1 production at 3 h and TNF-α, CXCL1, IL-6, and IL-10 at 24 h. Of note, levels of IL-6 and IL-10 at 3 h were below the detectable limit in baseline conditions, as were other cytokine levels in the panel.

### Statistical Analyses

To determine appropriate sample size, statistical power was calculated using G^*^Power 3.1.9.7 (Institut für Psychologie, Kiel, Germany) (74), using estimated means from previous studies (33, 42). Here, we prepared an n of three per treatment group, whereby each n represents isolated PBMCs from three animals pooled into one sample. Comparisons to determine statistical significance, defined as *p* < 0.05, were performed within Prism 7.05 (GraphPad software, San Diego, CA, USA). For comparisons between saline and methadone PMBC secretion, a Student *t*-test was used to determine significance. For statistical analysis of the dose response to LPS, a one-way analysis of variance (ANOVA) with Bonferroni multiple comparisons was performed. To make comparisons between treatments (baseline secretion vs. LPS-stimulated secretion) and to make comparisons between time points (3- vs. 24-h secretion), two-way ANOVA was performed with Bonferroni *post-hoc* analyses.

## Results

### Methadone Alters Baseline Peripheral Immune System Signaling

**Figure 2:**
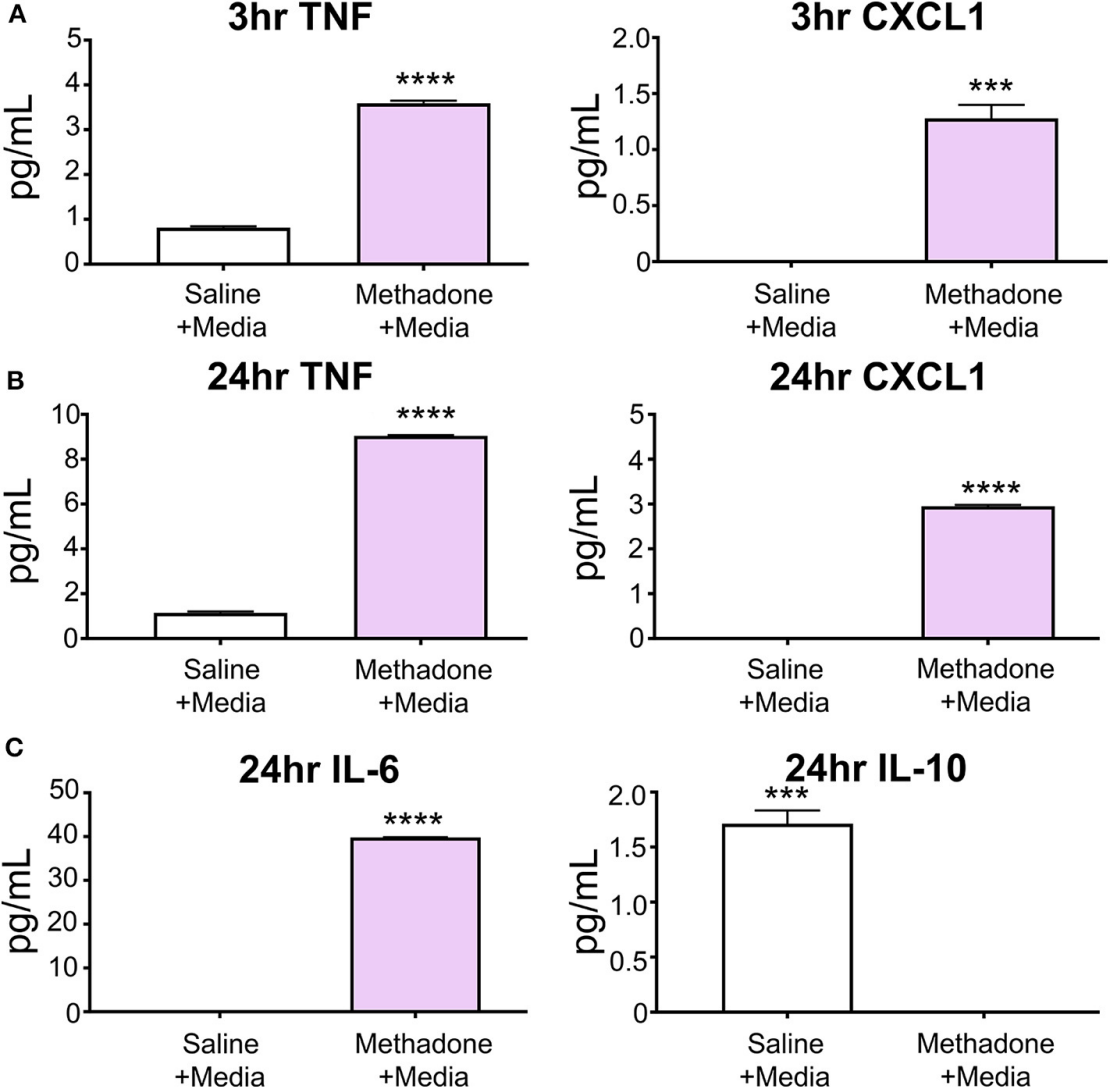
At baseline, PBMCs from methadone-exposed pups demonstrated dysregulated production of inflammatory signaling molecules. **(A)** At 3 h following isolation from P7 pups and plating in media alone, PBMCs from methadone-exposed pups produced significantly increased levels of TNF-α and CXCL1, compared to PBMCs from saline-exposed control PBMCs. **(B)** After 24 h, TNF-α and CXCL1 levels from methadone PBMCs compared to saline were further augmented. **(C)** Additionally, methadone PBMCs produced significantly higher levels of IL-6 and significantly lower levels of IL-10, compared to saline controls (*n* = 3 per treatment group, ***p < 0.001, ****p < 0.0001).

At 3 h, unstimulated PBMCs from methadone-exposed pups produced significantly augmented levels of the inflammatory cytokine TNF-α (methadone: 3.589 pg/mL, saline: 0.8142 pg/mL, *t*-test, *p* < 0.0001), as well as CXCL1 (methadone: 1.280 pg/mL, saline: 0.0, *t*-test, *p* = 0.0004) (Figure 2A).

After 24 h, the level of TNF-α secreted by saline PBMCs was 41% increased from levels measured at 3 h, whereas methadone PBMCs demonstrated a 152% increase from levels measured at 3 h (two-way ANOVA, *p* < 0.0001). Increased production of TNF-α by methadone PBMCs at 24 h (methadone: 9.047 pg/mL, saline: 1.148 pg/mL, *t*-test, *p* < 0.0001) was accompanied by increased secretion of CXCL1 by methadone PBMCs relative to saline PBMCs (methadone: 2.950 pg/mL, saline: 0.0 pg/mL, *t*-test, *p* = 0.0004) (Figure 2B). By 24 h, CXCL1 secretion by methadone PBMCs was 130% increased from measurement at 3 h (*t*-test, *p* < 0.0002), whereas levels of CXCL1 production by saline PBMCs remained below detectable levels at both the 3- and 24-h time points. Moreover, after 24 h in culture, methadone PBMCs demonstrated additional evidence of dysregulated immune signaling, evident by increased IL-6 (methadone: 39.82 pg/mL, saline: 0.0 pg/mL, *t*-test, *p* < 0.0001) and decreased IL-10 expression (methadone: 0.0 pg/mL, saline: 1.712 pg/mL, *t*-test, *p* = 0.0001) (Figure 2C). Together, these data show significantly altered baseline production of inflammatory signaling molecules by methadone-derived PBMCs at 3 and 24 h compared to controls.

### Methadone Primes the Peripheral Immune System Toward Hyperreactivity

**Figure 3:**
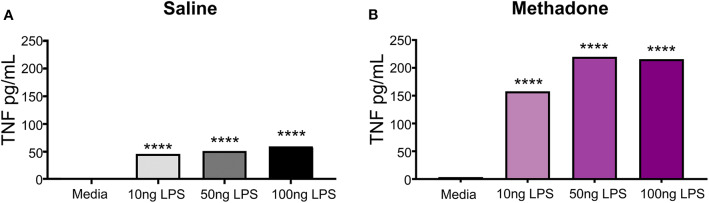
Peripheral blood mononuclear cells isolated from P7 pups exhibit LPS induced dose-responsive increases in TNF-α production. **(A)** Increasing doses of LPS (10, 50, or 100 ng/mL) elicited significantly augmented secretion of TNF-α from saline and **(B)** methadone PBMCs, compared to PMBCs in media alone (*n* = 3 per treatment group, 1 way ANOVA, ****p < 0.0001).

To illuminate potential discrepancies in reactivity to immune stimulus between treatment groups, PBMCs from methadone and saline exposed pups were challenged with LPS *in vitro*. By increasing the dose of LPS from 10, to 50, to 100 ng/mL, we observed a dose-dependent increase in TNF-α secretion from both saline and methadone PBMCs, compared to PMBCs in media alone (one-way ANOVA, *p* < 0.0001) (Figure 3). In this assay, addition of media alone resulted in levels of TNF-α production that were not statistically different from baseline levels of TNF-α measured at 3 h, as reported earlier in this study (one-way ANOVA, *p* > 0.05). With each dose of LPS, PBMCs derived from methadone-exposed animals produced greater levels of TNF-α compared to saline PBMCs (one-way ANOVA, *p* < 0.001). As 100 ng/mL LPS elicited a significant response in PBMCs from both methadone- and saline-exposed pups, we stimulated a separate cohort of PBMCs with this dose and measured cytokine and chemokine levels at 3 and 24 h.

**Figure 4:**
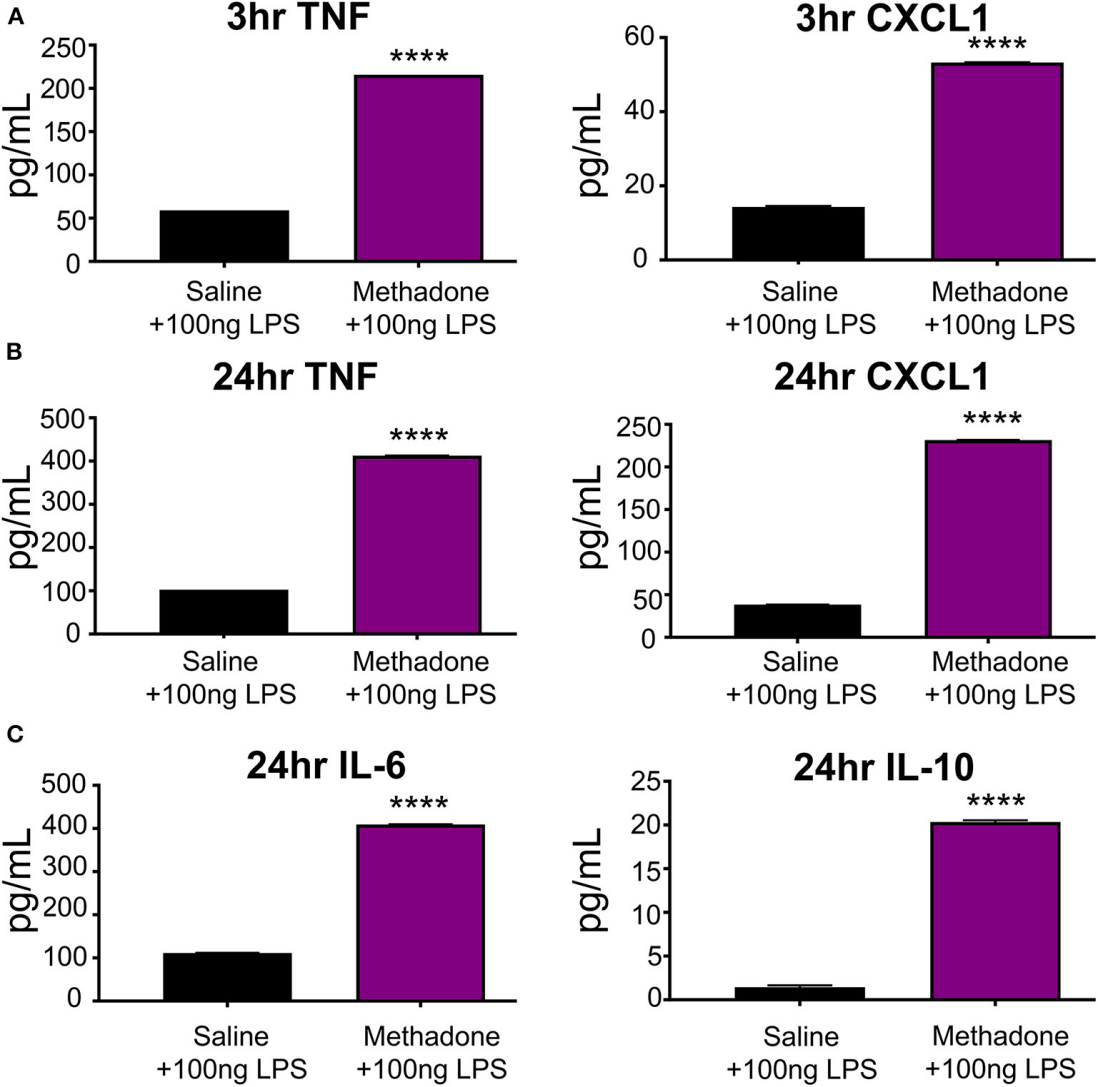
Stimulation with LPS revealed hyperreactivity of PBMCs from methadone-exposed pups. **(A)** At 3 h, TNF-α and CXCL1 production by PBMCs from P7 methadone-exposed pups was significantly increased compared to PBMCs from saline exposed controls. **(B)** After 24 h, TNF-α and CXCL1 levels from methadone PBMCs were significantly increased compared to saline levels. **(C)** Additionally, production of IL-6 and IL-10 by methadone PBMCs was significantly augmented, compared to saline controls (*n* = 3 per treatment group, unpaired *t*-test, ****p < 0.0001).

At 3 h following stimulation with 100 ng/mL LPS, TNF-α (methadone: 215.8 pg/mL, saline: 59.08 pg/mL, *t*-test, *p* < 0.0001) and CXCL1 (methadone: 53.29 pg/mL, saline: 14.33 pg/mL, *t*-test, *p* < 0.0001) production by PBMCs from P7 methadone-exposed pups was increased compared to PBMCs from saline-exposed controls, representing a 265 and 272% increase, respectively (Figure 4A). Compared to baseline levels of unstimulated PBMCS, LPS-stimulated PBMCs derived from both saline and methadone animals produced significantly increased levels of TNF-α and CXCL1 3 h (two-way ANOVA, *p* < 0.0001).

Following 24 h of LPS stimulation, levels of TNF-α (methadone: 412.6 pg/mL, saline: 102.8 pg/mL, *t*-test, *p* < 0.0001) and CXCL1 (methadone: 231.6 pg/mL, saline: 38.34 pg/mL, *t*-test, *p* < 0.0001) produced by methadone PBMCs were increased by 301 and 504%, respectively, compared to levels from saline PBMCs (Figure 4B). Compared to measurements taken at 3 h following LPS stimulation, TNF-α secretion at 24 h increased by 74% in saline PBMCs, whereas methadone PBMCs demonstrated a 91% increase (two-way ANOVA, *p* < 0.0001). A similar pattern arose with CXCL1 production. By 24 h, CXCL1 production by saline PBMCs increased 164% from levels measured at 3 h, whereas CXCL1 produced by LPS-challenged methadone PBMCs rose 334% (two-way ANOVA, *p* < 0.0001). At 24 h, we also found significantly increased levels of IL-6 (methadone: 409.4 pg/mL, saline: 111.7 pg/mL, *t*-test, *p* < 0.0001) and IL-10 (methadone: 20.35 pg/mL, saline: 1.442 pg/mL, *t*-test, *p* < 0.0001) from methadone PBMCs, compared to saline controls (Figure 4C).

## Discussion

As the opioid crisis continues to grow, increasing numbers of pregnant women and infants are affected. While mounting evidence indicates that prenatal opioid exposure is associated with significant and long-lasting neurological injury (17–19, 25), information on the pathophysiology of opioid exposure during the perinatal period is limited. Increasing our understanding of the cellular and molecular mechanisms that are impacted in circumstances of *in utero* opioid exposure is important for the development and implementation of informed clinical practices, in addition to the improvement of therapeutic options to support opioid-exposed infants.

Using the same model of perinatal opioid exposure employed in the current study, we previously provided evidence of neuroinflammation, microstructural brain injury, persistent cognitive deficits, and peripheral immune activation following perinatal opioid exposure (42). Here, we expand our understanding of the systemic immune dysfunction through an in-depth characterization of peripheral immune cell activity and reactivity following perinatal methadone exposure. Utilizing a clinically applicable *in vitro* cell culture protocol and translational multiplex immunoassay inflammatory biomarker panel (36, 37, 75–80), we found that at baseline PBMCs derived from methadone-exposed P7 pups hypersecreted proinflammatory molecules. Consistent with our previous findings that PBMCs derived from methadone-exposed rats secreted increased levels of TNF-α at 3 and 24 h after collection (42), here, we demonstrate that methadone PBMCs also secrete elevated levels of CXCL1 at 3 h. Increased expression of TNF-α and CXCL1 relative to PBMCs derived from saline-exposed controls was still evident at 24 h, demonstrating sustained peripheral immune activation of methadone-derived PBMCs at P7. Interestingly, after 24 h in culture, we observe additional evidence of dysregulated immune signaling with elevated IL-6 and diminished IL-10 in methadone-exposed animals relative to saline controls, in addition to the increased TNF-α and CXCL1 levels from methadone PBMCs. Despite probing for the presence of IL-6 and IL-10 at 3 h, the levels from both saline- and methadone-derived PBMCs remained below the detectable limits of the MECI. In summary, this evidence of elevated PBMC baseline production of known proinflammatory molecules TNF-α, CXCL1, and IL-6, with decreased levels of anti-inflammatory IL-10 from methadone-exposed animals, likely contributes to a proinflammatory environment within the systemic circulation (81).

To further characterize altered immune function, we stimulated cultured PBMCs with 100 ng/mL of LPS to represent an immune challenge and then quantified the chemokine and cytokine profile signature. While the addition of 100 ng/mL of LPS is a supraphysiological dose, it was formulated in consideration of our previous work (41, 42) and based on the dose response measured in TNF-α secretion to 10, 50, and 100 ng/mL of LPS that we performed in this study. We demonstrate that 100 ng/mL produces a robust PBMC response in the *in vitro* PBMC culture assay we performed, allowing us to examine chemokine and cytokine production by PBMCs from both saline and methadone treatment groups. Following 3 h of LPS stimulation, we detected levels of TNF-α and CXCL1 that were elevated from baseline in both methadone- and saline-exposed groups. Lipopolysaccharide-stimulated methadone PBMCs produced significantly more TNF-α and CXCL1 compared to PBMCs from stimulated saline control PBMCs. At 24 h, hyperreactivity of methadone PBMCs was evident from significantly elevated levels of TNF-α, CXCL1, IL-6, and IL-10 compared to saline controls. Taken together, these data provide evidence of increased sensitivity and a priming effect to subsequent inflammatory challenge in PBMCs from methadone-exposed pups. This suggests a mechanism of deleterious feed-forward inflammatory pathophysiology and fetal programming of immune system activation induced by methadone. Indeed, immune plasticity altered by methadone exposure may have long-lasting effects on the inflammatory responses of circulating leukocytes later in life. Future studies that assess the secretome and reactivity of PBMCs derived from opioid-exposed subjects at later developmental time points beyond P7 would be important to answer these questions.

Importantly, our *in vitro* approach allowed us to study PBMC responsiveness and sensitivity in isolation of potential confounders such as Toll-like receptor–stimulating agents in the peripheral circulation (82). Our *in vitro* data showing increased proinflammatory signaling at baseline from methadone-exposed PBMCs, without any immune stimulation, are distinct to this paradigm of perinatal injury and highlight this *in vitro* assay for use as a potential biomarker. The *in vitro* LPS challenge we perform here is a method that has been used clinically in children with developmental disorders and brain injury (35, 37). For instance, in a clinical study of children born preterm with cerebral palsy (37) and preclinical model reminiscent of preterm CNS injury (41), PBMCs were assayed consistent with the *in vitro* approach employed in the current study. Interestingly, in these studies, baseline secretion of PBMCs did not differ between treatment groups (37, 41). Only after stimulation with LPS did appreciable differences in PBMC chemokine and cytokine production appear in subjects with cerebral palsy (37), similar to preclinical studies (41, 45, 73). Perinatal insult–specific PBMC properties, revealed using this *in vitro* approach, support the potential use of secreted protein profiles from isolated PBMCs as a biomarker to discern distinct pathologies and potentially guide clinical treatment. Indeed, elucidating these profiles of immune signaling molecules holds potential for use as a biomarker to determine vulnerability to sustained peripheral immune hyperreactivity. Specifically, biomarkers in neonates could provide estimation of extent of immune system abnormalities and CNS injury and provide pharmacodynamic support to guide duration or degree of treatment for neonatal opioid withdrawal syndrome or supportive care in neonatal intensive care units. In this context, durable changes in PBMC reactivity may be an effective biomarker, and clinical utility may prove high given the ease of access to these cells and well-defined clinical stimulation protocols (37, 43). However, while these *in vitro* PBMC assays are clinically relevant, they are distinctly different than studying the complex, multidimensional *in vivo* response to inflammation, sepsis, and systemic sensitization catalyzed by an LPS challenge. Unquestionably, further study is required to validate how circulating leukocytes respond to LPS immune challenges *in vivo* and in the context of complex inflammatory networks, systemic circulating factors, and all cells that express TLR4.

Peripheral blood mononuclear cell hypersecretion of proinflammatory molecules and PBMC hyperreactivity resultant of gestational opioid exposure have important implications for the developing CNS. Our previous preclinical report strongly implicates brain injury secondary to opioid-induced systemic and neuroinflammation (42). In alignment with the aforementioned study, we now provide evidence of PBMC hypersection and hyperreactivity, which could contribute to increased systemic inflammation during the term equivalent developmental time point, coinciding with the brain growth spurt, peak myelination and gliogenesis, and astrocyte production (60, 63, 65–69, 83). Increased chemokine and cytokine production by PBMCs during the perinatal period jeopardizes proper neural cell development and circuitry maturation. Indeed, inflammation during perinatal development results in lasting neurological impairment (77, 80, 84, 85). While future studies are needed to clarify if methadone elicits PBMC systemic inflammation via PBMC activation throughout the methadone exposure, it is well-established that elevated levels of circulating inflammatory proteins during later stages in brain development (late third trimester, term equivalent) are associated with brain injury, characterized by increased structural and functional neurological deficits (80, 84, 86–89). Specifically, in a recent study of systemic TNF-α inhibition in preterm fetal sheep exposed to LPS-induced inflammation, researchers identified circulating TNF-α as a critical contributor to neuroinflammation and pathogenesis of impaired neurodevelopment (90). Cytokine and chemokines produced by circulating leukocytes are able to cross the blood–brain barrier via selective transporters (91). Furthermore, as in the specific case of TNF-α, increased levels can contribute to impairment of the blood–brain barrier function (92–94), allowing for increased proinflammatory molecule access to the developing CNS. Opioids are able to cross from maternal circulation through the placenta to fetal circulation owing to their low molecular weight, moderate lipid solubility, and low protein binding (95). Once in fetal circulation, opioids are able to cross the fetal blood-brain barrier by means of numerous transporters (96, 97). Thus, not only are developing neural cells and circuitry exposed to elevated levels of proinflammatory molecules in the context of opioid exposure, but they are directly exposed to opioids as well. In the developing CNS, neurons in addition to oligodendrocytes, astrocytes, and microglia express opioid receptors (98). Intriguingly, oligodendrocytes express opioid receptors in a maturation-dependent manner, in which immature stages of oligodendrocytes have increased opioid receptor expression (98), rendering this population more vulnerable to opioid exposure. Ultimately, exposure to opioids in conjunction with the proinflammatory profile produced by opioid exposure characterized in the current investigation could contribute to observed brain injury in preclinical studies (42), as well as clinical studies indicating particular vulnerability to major white matter tracts in infants exposed to opioids during brain development (20, 22, 24, 25).

Similar to the CNS, the immune system develops and matures over the course of gestation and the perinatal period. Dysregulated chemokines and cytokine production, and changes in immune cells themselves, culminate in impaired immune function that can last decades (35, 37). Similar to neural cells, leukocytes are uniquely responsive to their environment. Indeed, immune plasticity altered by prenatal insults may have long-term effects on the inflammatory responses of circulating leukocytes, which may serve as a biomarker of persistent or prior neuroinflammation and brain injury (99, 100). Infants exposed to intrauterine inflammation are at an increased risk of neurodevelopmental disorders (101). Notably, newborns that have elevated levels of biomarkers of systemic inflammation on two occasions 1 week apart are at a higher risk of brain injury and impaired neurodevelopment (77, 80, 84) Thus, understanding the homeostatic regulation of central and peripheral inflammatory cells in infants following opioid exposure, and the long-term consequences of their dysregulation, is essential (102). Significantly, an increase in chemokines/cytokines can contribute to perinatal brain injury by multiple overlapping mechanisms, including direct initiation of programed cell death pathways, microglial activation, immune cell recruitment, mitochondrial damage, and endoplasmic reticulum stress (85, 103, 104).

There are important limitations to this present study. For instance, here PBMCs were isolated from pooled peripheral blood from term equivalent male and female rats, limiting the ability to elucidate differences between individual animals and between male and female rat pups. Evidence from studies examining PBMCs isolated from adult humans suggests that sex differences in stimulated PBMC properties and secretion exist (105–107). While sex differences in secretion of PBMCs isolated at neonatal time points are not well-defined (35, 37), evidence exists demonstrating sex-specific differences in brain inflammation following circulating myeloid cells depletion in neonatal mice (108) and that inflammatory responses following immune cell activation in the immature brain differ between males and females, as reviewed by Mallard et al. (109). Thus, separate pooling of males and female peripheral blood from P7 pups for sex-specific analysis represents an important future direction. Additionally, although pooling of blood from multiple P7 rat pups was necessary in these experiments to collect an adequate PBMC fraction following differential centrifugation, analysis at later time points with larger animals would not require pooling, allowing for analysis of individual animal PBMC secretion and reactivity. Peripheral blood mononuclear cells represent a heterogeneous population of mononuclear cells in the peripheral circulation composed of T cells, T regulatory cells, T helper cells, B cells, and natural killer/dendritic cells/monocytes (110). Undoubtedly, flow cytometric studies beyond the scope of the present investigation are needed to define the precise immune cell population composition of PBMCs isolated from animals exposed to opioids during development.

In the current study, pregnant ram dams were implanted with methadone administering osmotic minipumps on E16 prior to complete oligodendrocyte, microglial, and astrocyte maturation (60), limiting rat pup opioid exposure to E16 through P7 when PBMCs were collected. This prenatal and postnatal opioid exposure paradigm accomplishes opioid exposure up until the equivalent end of the human third trimester. Future studies should now aim to commence opioid exposure from the onset of pregnancy (E0), thereby encompassing the entirety of brain and immune development.

In conclusion, we provide evidence in support of a systemic inflammatory response to perinatal opioid exposure, characterized by immune cell reprogramming and priming. This evidence may in part contribute to the neurological injury following developmental opioid exposure characterized in our previous preclinical study (42). The current study with the study by Jantzie et al. (42) joins a host of new and intriguing investigations that link developmental neurological injuries including cerebral palsy (37) and Down syndrome (35) with underlying systemic inflammation resultant of abnormal PBMC activity. Treatments that reduce inflammation or support developing neural cells in the context of inflammation could rescue the poor neural outcomes observed in preclinical and clinical investigations of perinatal opioid exposure. Our future studies will aim to identify appropriate therapies that target these proinflammatory mechanisms underlying brain injury associated with *in utero* opioid exposure (33, 111–114).

## Data Availability Statement

The datasets generated for this study are available on request to the corresponding author.

## Ethics Statement

The animal study was reviewed and approved by the Institutional Animal Care and Use Committee (IACUC) at the University of New Mexico Health Sciences Center.

## Author Contributions

LJ conceptualized the hypothesis and supervised the experiments. JN, JM, YK, SR, and LJ designed and performed the experiments. SR and LJ interpreted the data. JN, LJ, and SR wrote the manuscript. All authors contributed to manuscript revision and approved the final version.

## Conflict of Interest

The authors declare that the research was conducted in the absence of any commercial or financial relationships that could be construed as a potential conflict of interest.
